# Gut microbiota characteristics of gastric cancer patients across distinct pathological stages and their associations with tumor and paratumor mucosal microbiota

**DOI:** 10.3389/fcimb.2026.1897107

**Published:** 2026-09-17

**Authors:** Guoqing Qi, Zhiping Wu, Juanjuan Chen, Dekui Zhang, Qi Wang

**Affiliations:** 1 The Second Clinical Medical College, Lanzhou University, Lanzhou, China; 2 Department of Gastroenterology, Lanzhou University Second Hospital, Lanzhou, China; 3 Cuiying Biomedical Research Center, The Second Hospital & Clinical Medical School, Lanzhou University, Lanzhou, China

**Keywords:** gastric cancer, gut microbiome, intratumoral microbiome, microbial dysbiosis, paracancer microbiome

## Abstract

Gastric cancer (GC) is closely associated with microbial dysbiosis; however, the spatial–temporal characteristics of the gut, paracancerous, and intratumoral microbiomes, as well as their interrelationships during tumor progression, remain insufficiently defined. This study aimed to characterize these microbiomes in 85 GC patients from Northwest China using shotgun metagenomic sequencing and functional annotation. Clinical samples included feces, tumor tissues, and paratumor mucosal tissues (≥5 cm from the tumor margin), enabling comprehensive profiling of microbial composition at the phylum, genus, and species levels, as well as functional pathway analysis. The results showed that the microbial community was dominated by Firmicutes and Bacteroidetes (combined relative abundance >75%), maintaining structural stability across tumor stages II–IV (Stage I n=2 is only descriptively reported and excluded from formal statistical testing), thereby supporting the “core microbiota resilience” hypothesis. PERMANOVA revealed that tumor stage had a statistically significant yet modest effect on community structure (R² = 0.039, P = 0.032). MaAsLin2 identified stage-associated differential genera: *Roseburia* and *Megamonas* decreased with advancing stage, whereas *Lactobacillus* and *Enterobacter* were enriched in advanced stages (III/IV). Functional pathway analysis revealed stage-specific metabolic remodeling: Stage II was enriched in DNA repair, glycolysis, aromatic amino acid, and nucleotide biosynthesis pathways; Stage IV showed enrichment in protein deamination/demethylation, pyruvate fermentation, and coenzyme metabolism pathways. Correlation analysis further revealed a characteristic pattern of “pathogen enrichment and beneficial bacteria depletion.” *Fusobacterium nucleatum* and *Helicobacter pylori* exhibited strong positive correlations with GC, whereas short-chain fatty acid–producing bacteria such as *Roseburia* and *Faecalibacterium prausnitzii* showed significant negative correlations, particularly in paracancerous tissues. We established multi-layered taxonomic correlation profiles based on intra-cohort microbial shifts, in which Bacteroides stercoris and Bifidobacterium pseudocatenulatum exhibited the most pronounced stage-related abundance changes in the fecal microbiota across tumor stages. Overall, this study systematically delineates the structural stability, stage-specific functional remodeling, and interrelated dynamics of gut, paracancerous, and intratumoral microbiomes in gastric cancer. These findings provide descriptive baseline data of cross-compartment microbiome variation within gastric cancer patients across tumor stages, which only deliver preliminary correlative clues of GC-related microbial shifts and require further multi-cohort verification with non-cancer control populations.

## 1 Background

Gastric cancer (GC) is the fifth most common malignant tumor globally and the fourth leading cause of cancer-related deaths worldwide (Sung et al., 2021). Its development is associated not only with host genetics, diet, environmental factors, and *Helicobacter pylori* (*H. pylori*) infection, but also with alterations in the composition and function of both the gastric and distal gut microbiota (Meng et al., 2018; Chang et al., 2024). With the widespread application of high-throughput sequencing, metagenomics, and multi-omics integration technologies, increasing attention has been directed toward the bidirectional regulatory role of the gastrointestinal microbiota and the “gut–stomach axis” in the initiation, progression, treatment response, and prognosis of GC (Ferreira et al., 2018; Chang et al., 2024).

Before the discovery of *H. pylori*, the stomach was long considered a sterile organ. To date, *H. pylori* remains the only bacterium classified as a Group I carcinogen (Warren and Marshall, 1983). Earlier research on gastric microbes primarily focused on *H. pylori*, particularly because *H. pylori* infection is recognized as a major risk factor for GC (Collatuzzo et al., 2021; Mendes-Rocha et al., 2023). *H. pylori* infects approximately 50% of the global population; however, fewer than 3% of infected individuals ultimately develop GC, and eradication of *H. pylori* does not completely eliminate the risk of gastric cancer development (Mirzaei et al., 2021; Kumar et al., 2020).

Owing to difficulties in culturing and identifying bacteria from gastric juice, the role of the microbiota in GC development was underestimated for many years. Recent advances in genomic technologies have enabled improved characterization of the gastric microbiome, thereby strengthening the evidence base and promoting deeper investigation into gastric dysbiosis and carcinogenesis (Sung et al., 2016). Contrary to the earlier belief that the acidic environment of the human stomach precluded colonization by microbes other than *H. pylori* (Devi et al., 2021; Zhou et al., 2024), sequencing-based studies have revealed a diverse gastric microbiota and linked GC to alterations in microbial diversity and abundance (Zhou et al., 2024). Studies indicate that, in addition to *H. pylori*, certain microbial species, such as lactobacilli, can survive in the acidic and pepsin-rich gastric environment (Ait Seddik et al., 2017; Johansson et al., 2013). Nevertheless, microbial biomass in the stomach (10²–10^4^ colony-forming units [CFU]/mL) remains substantially lower than that in the intestine (10^5^–10^12^ CFU/mL) (Cella et al., 2024; Aron-Wisnewsky et al., 2012).

From a spatial perspective, marked differences exist in microbial distribution among the cardia, gastric body, gastric antrum, and tumor center, margin, and paratumor mucosal tissue. GC tissues exhibit enrichment of multiple genera, including *Acinetobacter*, *Fusobacterium*, *Lactococcus*, *Lactobacillus*, *Peptostreptococcus*, *Prevotella*, and *Streptococcus*, whereas paratumor mucosal tissue is predominantly colonized by *Helicobacter* (Liu et al., 2022; Dai et al., 2021). Recent multicenter 16S rRNA and metatranscriptomic studies have further shown that, besides *H. pylori*, GC tissues are enriched in anaerobes such as *Porphyromonas*, *Peptostreptococcus*, *Streptococcus*, and *Fusobacterium*, while *Lactobacillus* is reduced, forming a GC-associated microbiota signature. These bacterial genera may contribute to the atrophy–intestinal metaplasia–dysplasia cascade by activating the NF-κB/IL-23/IL-17 axis, inducing reactive oxygen species and N-nitroso compound production, and promoting DNA methylation and histone modifications. In addition, single-cell RNA-seq combined with microbiome analysis has shown that *Fusobacterium nucleatum* enriched in GC tissues downregulates CD8^+^ tissue-resident memory T cells (T_RM_) and enhances PD-L1 expression, thereby facilitating immune escape (Zeng et al., 2024). Accumulated DNA damage and dysregulated homologous recombination (HR) repair represent critical molecular hallmarks during gastric carcinogenesis and also determine the therapeutic response to DNA-damage-targeted agents. Poly(ADP-ribose) polymerase inhibitors (PARPi) display limited efficacy in most gastric tumors owing to the low frequency of intrinsic HR deficiency. Pharmacological disruption of Arp2/3 complex function can impair cellular HR capacity and produce synthetic-lethal effects together with PARP inhibitors, offering a promising strategy to expand PARPi application in HR-proficient gastric cancer (Li et al., 2026). Mounting evidence suggests that gut-gastric microbiota can trigger DNA lesions and modulate host DNA-repair signaling, which may indirectly shape tumor HR status and drug sensitivity.

Temporally, most studies report that gastric microbiota diversity or abundance decreases with GC progression, with concurrent reductions in microbial richness and diversity observed in oral and fecal samples (Yu et al., 2021; Zeng et al., 2024). However, substantial heterogeneity exists among microbial communities, which can affect the consistency of study findings. For example, gut microbiota composition shows pronounced geographical variation, particularly in the distribution of *Bacteroidetes* and *Firmicutes*. In samples from the United States, *Firmicutes* predominate, whereas *Bacteroidetes* are more abundant in Chilean samples. Calculation of the Firmicutes-to-Bacteroidetes (F:B) ratio revealed the highest ratio in U.S. samples (4.15) and the lowest in Chilean samples (Kumar and Bhadury, 2023). Studies in Asian populations likewise indicate that regional differences influence the relative abundance of *Bacteroidetes* and *Firmicutes*, likely reflecting variations in diet, cultural practices, and environmental exposures (Taha et al., 2023). In parallel, the gastric bacterial community may expand and undergo compositional changes during medication use or carcinogenic processes; for example, acid-suppressive agents such as proton pump inhibitors raise gastric pH above 6, thereby promoting microbial growth (Zhu et al., 2024).

Although microbial communities of the lower gastrointestinal tract may exert a more indirect influence on gastric carcinogenesis, their association with GC cannot be disregarded (Zeng et al., 2024). *Streptococcus*, *Fusobacterium*, *Prevotella*, and *Veillonella* are enriched in fecal samples from patients with GC (Yu et al., 2021; Zhou et al., 2022; Zhang et al., 2022; Qi et al., 2019; Zhang et al., 2021). At the species level, fecal abundances of *Streptococcus anginosus* and *Streptococcus constellatus* are increased (Zhou et al., 2022). Furthermore, GWAS-based Mendelian randomization analyses suggest that genetically predicted abundances of taxa such as *Clostridium sensu stricto 1*, *Actinomycetales*, and *Negativicutes* are negatively associated with GC risk (OR 0.54–0.86), whereas increased abundances of *Roseburia* and the *Eubacterium brachy* group are positively associated with risk, supporting a potential causal relationship between GC and specific gut microbes (Chang et al., 2024).

However, research examining the relationship between gastric and gut microbiota in GC patients remains limited. In this study, we aim to investigate the gastric and gut microbiota in patients with different stages of GC from Northwest China using shotgun metagenomic sequencing. This study investigated changes in microbial taxon abundance within fecal, tumor, and paratumor compartments, as well as their cross-compartment associations, to provide a theoretical foundation for understanding their roles in gastric cancer pathogenesis and progression.

## 2 Materials and methods

### 2.1 Study design and ethics statement

This study was designed to characterize the gut microbiome, paracancerous tissue microbiome, and intratumoral microbiome in patients with gastric cancer (GC) from Northwest China using shotgun metagenomic sequencing. The samples and clinical information used in this study were obtained under written informed consent and approved by the Ethics Committee of Lanzhou University Second Hospital (Approval No.2021A-459). The study was conducted in accordance with the Declaration of Helsinki (2013 revision).

### 2.2 Subject recruitment and sample collection

#### 2.2.1 Patient recruitment and criteria

A total of 85 patients who underwent radical gastrectomy for gastric cancer at Lanzhou University Second Hospital were enrolled (January 2021–December 2023). Eligible participants met the following criteria:

Age range between 20 and 85 years (actual range: 24–83 years).

No history of chronic infectious diseases (e.g., tuberculosis, hepatitis), metabolic disorders, such as diabetes mellitus and obesity with BMI ≥30 kg/m², or other known malignancies.

No intake of antibiotics, probiotics, prebiotics, or medications known to influence the gut microbiota (e.g., proton pump inhibitors, non-steroidal anti-inflammatory drugs) for at least one month prior to sample collection.

No consumption of fermented foods (e.g., pickles, yogurt, fermented bean curd) for at least one month.

Weaned, with a habitual diet based on rice, cooked wheaten foods, meat, and vegetables, and a daily milk intake of less than 300 mL.

Provision of complete phenotypic information and written informed consent.

Pathologically confirmed gastric cancer (excluding gastroesophageal junction cancer) with clear tumor staging according to the 8th edition of the AJCC TNM Classification.

#### 2.2.2 Exclusion criteria

Concurrent inflammatory bowel disease, irritable bowel syndrome, or other gastrointestinal disorders.
History of gastrointestinal surgery (except for the current gastrectomy).
Severe hepatic, renal, or cardiac dysfunction.
Pregnancy or lactation.

#### 2.2.3 Phenotypes and sample collection

Basic demographic and clinical information, including age, sex, body mass index (BMI), ECOG performance status, Charlson Comorbidity Index (CCI) score, nationality, tumor stage (AJCC 8th edition), primary tumor location, Lauren classification, surgical procedure, and chemotherapy history, were collected (**Supplementary Tables S1A**).

Fecal sample collection: Fresh fecal samples (5–10 g) were collected from each participant 1–3 days prior to surgery, immediately placed into sterile cryotubes, snap-frozen in liquid nitrogen within 30 minutes, and stored at −80 °C until further processing.

Tissue sample collection: Postoperative tumor tissue (≥100 mg) and paratumor mucosal tissue (≥100 mg, located ≥5 cm from the tumor margin and pathologically confirmed to be free of tumor cells) were collected under sterile conditions, rinsed with pre-cooled sterile phosphate-buffered saline (PBS, pH 7.4) to remove blood and debris, snap-frozen in liquid nitrogen, and stored at −80 °C.

Sample quality control: Fecal samples showing visible contamination (e.g., urine or soil) were excluded. Tissue samples were verified by hematoxylin–eosin (H&E) staining to confirm ≥70% tumor cell content in tumor tissues and the absence of tumor cell infiltration in paracancerous samples. During gastrectomy sampling, we collected environmental blank swabs (operating table surface, surgical glove exterior, empty sterile tube blanks) as parallel negative controls, extracted DNA following identical tissue protocols.

### 2.3 Shotgun metagenomic sequencing and data analysis

#### 2.3.1 DNA extraction, library preparation, and sequencing

##### 2.3.1.1 DNA extraction

Total genomic DNA was extracted from fecal samples (200 mg) using the QIAamp PowerFecal Pro DNA Kit (Qiagen, Germany) and from tissue samples (50 mg) using the DNeasy Blood & Tissue Kit (Qiagen, Germany), according to the manufacturers’ protocols. For tissue samples, an additional mechanical lysis step (bead beating with 0.1 mm glass beads at 6500 rpm for 30 s × 3 cycles) was performed to enhance bacterial cell disruption.

##### 2.3.1.2 DNA quality control

DNA concentration was measured using a Qubit 4.0 Fluorometer (Invitrogen, USA), and purity was assessed by 1% agarose gel electrophoresis (A260/A280 ratio: 1.8–2.0; A260/A230 ratio: ≥1.5). DNA integrity was verified using an Agilent 2100 Bioanalyzer (Agilent Technologies, USA), Only DNA samples meet all the following criteria were selected for subsequent library construction: 1) A260/A280 ratio between 1.8 and 2.0; 2) intact main bands without noticeable smearing on the gel, along with a clear single high-molecular-weight band above 15 kb; 3) double-stranded DNA concentration ≥10 ng/μL and total DNA amount >100 ng. The DNA samples were classified as A,B,C, or D based on their contents and integrity, with A and B being directly usable for library construction and sequencing. Notably, all the samples except for negative controls (n=4) fell under classification A, indicating high-quality DNA (**Supplementary Table S3**).

##### 2.3.1.3 Library preparation

Metagenomic libraries were constructed using the TruSeq DNA PCR-Free Library Preparation Kit (Illumina, USA) with 1 μg of qualified DNA per sample. Libraries were size-selected for 350 bp inserts using AMPure XP beads (Beckman Coulter, USA) and quantified using Qubit 4.0 and the Agilent 2100 system.

##### 2.3.1.4 Sequencing

Paired-end sequencing (150 bp×2) was performed on the BGI-Seq500 platform (BGI-Shenzhen, China), with an average sequencing depth of 30 Gb per fecal sample and 20 Gb per tissue sample.

#### 2.3.2 Data processing and bioinformatics analysis

##### 2.3.2.1 Raw data filtering

Raw sequencing reads were processed using FASTP (v0.23.2) with the following parameters:

fastp -i raw_R1.fq -I raw_R2.fq -o clean_R1.fq -O clean_R2.fq \
-q 20 -u 50 -n 5 -l 50 --trim_front1 15 --trim_tail1 5 --trim_front2 15 --trim_tail2 5

Reads with >5 ambiguous bases, >50% of bases with a quality score ≤20, or length of 0 bp after trimming were discarded. Adapter sequences and low-quality bases at both ends were trimmed.

##### 2.3.2.2 Host sequence removal

Clean reads were aligned to the human reference genome (hg38) using Bowtie2 (v2.4.5). Reads mapped to the human genome (alignment rate >90%) were discarded, and unmapped reads were retained for downstream analysis (**Supplementary Table S4**).

##### 2.3.2.3 Taxonomic profiling

MetaPhlAn 4.0 was used with parameters -input_type fastq -ignore_viruses -ignore_eukaryotes -nproc 8 -t rel_ab. Taxonomic profiles were generated at the phylum, genus, and species levels.

##### 2.3.2.4 Functional profiling

HUMAnN 3 was run with the following parameters: -i input_clean_data -o output --threads 8 --memory-use maximum --remove-temp-output --taxonomic-profile metaPhlAn_output.tsv --pathabundance --pathcoverage. Functional pathways were annotated against the UniRef90 and KEGG (Kyoto Encyclopedia of Genes and Genomes) databases. Gene Ontology (GO) functional annotation was performed using eggNOG-mapper (v2.1.9).

#### 2.3.3 Diversity analysis

##### 2.3.3.1 α-Diversity

α-Diversity indices, including richness (observed genus/species taxonomic profiles), Shannon, Simpson, and Inverse Simpson indices, were calculated using the R package vegan (v2.6-4). Rarefaction curves were used to evaluate the sufficiency of the sequencing depth by randomly selecting six samples, subsampled the sequencing data from 1 to 100% of the original read depth, performed species annotation and diversity index calculations on each subsampled dataset, and plotted the relationship between sampling depth and observed diversity (**Supplementary Table S2**).

##### 2.3.3.2 β-Diversity

β-Diversity was calculated based on Bray-Curtis distance and Jaccard distance. Principal Coordinate Analysis (PCoA) and non-metric multidimensional scaling (NMDS) were performed using the *vegan* and *ape* (v5.7-1) packages.

##### 2.3.3.3 Group difference testing of taxon and functional pathway

Permutational multivariate analysis of variance (PERMANOVA) was performed using *adonis2* with the formula dist ~ tumor.staging and 999 permutations. The *envfit* test was used to assess the association between tumor stage and community structure.

MaAsLin2 was applied to identify bacterial genera and species whose relative abundances and differential functional pathways were significantly associated with tumor stage.

#### 2.3.4 Correlation analysis

SparCC and Spearman analysis was used to assess associations between microbial taxa (phylum, genus, species) and clinical parameters (tumor stage and Lauren type). Partial correlation analysis was performed using the *ppcor* package (v1.1) to adjust for confounding factors, including age, sex, and BMI. Correlation heatmaps and bubble plots were generated using *pheatmap* (v1.0.12) and *ggplot2* (v3.4.4), with significance markers (*P < 0.05, +P < 0.01).To mitigate compositional bias inherent in relative-abundance-based metagenomic profiles, SparCC and Spearman analysis with bootstrapped two-sided pseudo-p-value estimation was additionally performed.

#### 2.3.5 Statistical methods

Continuous variables (e.g., age, BMI, α-diversity indices) were presented as mean ± standard deviation (SD) and compared using the Mann–Whitney U test (two groups) or Kruskal–Wallis H test (multiple groups). Categorical variables (e.g., sex, tumor stage, Lauren type) were presented as counts and percentages and compared using Fisher’s exact test (n < 5) or the chi-square test (n ≥ 5). All statistical tests were two-tailed, with P < 0.05 considered statistically significant. Multiple comparisons were adjusted using the BH method for FDR control. Statistical analyses were performed using R (v4.2.1) and GraphPad Prism (v9.5.0). We applied R package decontam (Davies et al., 2018) for frequency-based contaminant identification. (**Supplementary Table S5**) PERMDISP test was performed alongside PERMANOVA to confirm inter-group community differences were not driven by uneven sample dispersion.

## 3 Results

### 3.1 Phenotypic analysis of the patients

This study recruited a total of 85 gastric-cancer patients, with a mean age of 58.4 ± 9.9 years, including 14 (16.5%) females and 71 (83.5%) males. Patients covered all tumor stages: stage I 2(2.4%), stage II 30(35.3%), stage III 24(28.2%) and stage IV 29(34.1%). The primary tumor sites were predominantly the cardia and fundus of stomach (49.4%). In total, 69.4% of patients received chemotherapy, and 30.6% were positive for *Helicobacter pylori* infection. Comprehensive baseline phenotypic characteristics are provided in **Supplementary Table S1A**. The sample-level Venn diagram (**Supplementary Figure 8**; **Supplementary Table S8**) illustrated the sample distribution across fecal, tumor, and paratumor groups.

### 3.2 Analysis of gut microbial taxonomic composition and functional pathways

MetaPhlAn 4.0 identified 10 phyla, 132 genera, and 352 species across the subjects. The α-diversity for species and genus was evaluated using the Shannon, Simpson, and Inverse Simpson indices. The Shannon, Simpson, and Inverse Simpson indices for species and genus were 2.94 ± 0.46, 0.89 ± 0.08, 11.70 ± 5.78, and 2.27 ± 0.44, 0.80 ± 0.13, 6.41 ± 2.80, respectively. *Firmicutes*, *Bacteroidetes*, *Actinobacteria*, and *Proteobacteria* were the four most abundant phyla (**Figure 1A**). The microbial community in the samples was dominated by *Firmicutes* and *Bacteroidetes*, with their combined relative abundance accounted for approximately 75%, consistent with the core compositional characteristics of the human gut microbiota. Other detected phyla included *Actinobacteria*, *Proteobacteria*, *Verrucomicrobia*, *Fusobacteria*, *Synergistetes*, *Tenericutes*, and *Euryarchaeota*. The relative abundance of each phylum ranged from 0% to 25%, constituting secondary components of the community. Overall, the phylum-level composition exhibited features of “high dominance and low diversity,” in which a small number of core phyla dominated the community structure while low-abundance phyla contributed to overall diversity. *Bacteroides*, *Bifidobacterium*, *Lactobacillus*, *Prevotella*, *Ruminococcus*, *Faecalibacterium*, *Blautia*, *Collinsella*, *Alistipes*, *Escherichia*, *Streptococcus*, *Parabacteroides*, *Lachnospiraceae_unclassified*, *Roseburia*, *Eubacterium*, *Dorea*, *Fusicatenibacter*, *Coprococcus*, *Anaerostipes*, and *Enterococcus* were the top 20 abundant genera (**Figure 1B**). Among these, *Bacteroides*, *Bifidobacterium*, *Lactobacillus*, *Prevotella*, *Ruminococcus*, *Blautia*, and *Faecalibacterium* were identified as core dominant genera. Their mean occurrence frequencies predominantly ranged between 0.6 and 0.95, indicating that these genera were widely distributed and relatively abundant across samples. Among the top 30 species (**Figure 1C**), those belonging to the genera *Bacteroides* and *Lactobacillus* were predominant. Representative species include: 1. *Bacteroides*: *Bacteroides caccae*, *Bacteroides dorei*, *Bacteroides thetaiotaomicron*, *Bacteroides stercoris*, *Bacteroides uniformis*, etc.; 2. *Lactobacillus*: *Lactobacillus reuteri*, *Lactobacillus salivarius*; 3. Other core species: *Faecalibacterium prausnitzii*, *Ruminococcus torques*, *Ruminococcus gnavus*, *Blautia obeum*, *Alistipes finegoldii*, etc. The mean occurrence frequencies of core species varied across taxa. *Escherichia coli* exhibited the highest occurrence frequency (0.95), indicating its widespread presence in the samples. Most dominant species showed occurrence frequencies ranging between 0.4 and 0.85, whereas a few low-abundance species (such as certain *Ruminococcus* species) had occurrence frequencies around 0.2, reflecting heterogeneity in species distribution across samples. GO functional pathways (**Figure 1D**: Top 30 abundant GO pathways) encompassed three major categories: Biological Process (BP), Molecular Function (MF), and Cellular Component (CC). Among these, MF-related pathways were the most numerous, followed by BP. Core functional pathways: 1. Molecular Function (MF): Primarily includes catalytic activity, transferase activity, hydrolase activity, kinase activity, metal ion binding, ATP binding, and GTP binding, reflecting the metabolic and catalytic capacity of the microbial community. 2. Biological Process (BP): Concentrates on processes such as carbohydrate metabolic processes, regulation of transcription (DNA-templated), translation, transmembrane transport, and DNA recombination/integration, involving core life processes related to metabolism and genetic information transfer. 3. Cellular Component (CC): Mainly includes ribosome, plasma membrane, cytoplasm, and integral components of membranes, indicating localization of functional pathways to core cellular structures. UniRef functional pathways (**Figure 1E**: Top 30 abundant UniRef pathways): Core metabolic pathways:1. Sugar metabolism: Glycolysis-related pathways (ANAGLYCOLYSIS-PWY: glycolysis III; PWY-1042: glycolysis IV) and the starch degradation pathway (PWY-6737: starch degradation V), serving as core pathways for energy metabolism. 2. Amino acid metabolism: Branched-chain amino acid biosynthesis (BRANCHED-CHAIN-AA-SYN-PWY), L-valine synthesis (VALSYN-PWY), L-isoleucine synthesis (ILEUSYN-PWY), L-lysine synthesis (PWY-2942, PWY-5097), and aromatic amino acid biosynthesis (COMPLETE-ARO-PWY), involved in the synthesis and metabolism of host-essential amino acids. 3. Nucleotide and coenzyme metabolism: tRNA charging (TRNA-CHARGING-PWY), *de novo* synthesis of purine and pyrimidine nucleotides (PWY-7221, PWY-7219, PWY-5686), coenzyme A biosynthesis (COA-PWY-1), and folate transformations (PWY-3841), ensuring substrates for genetic information transfer and metabolic coenzymes. 4. Cell wall synthesis: Peptidoglycan biosynthesis pathways (PEPTIDOGLYCANSYN-PWY, PWY-6385, PWY-6386, PWY-6387) and dTDP-β-L-rhamnose synthesis (DTDPRHAMSYN-PWY), involved in microbial cell wall construction and homeostasis maintenance.

**Figure 1:**
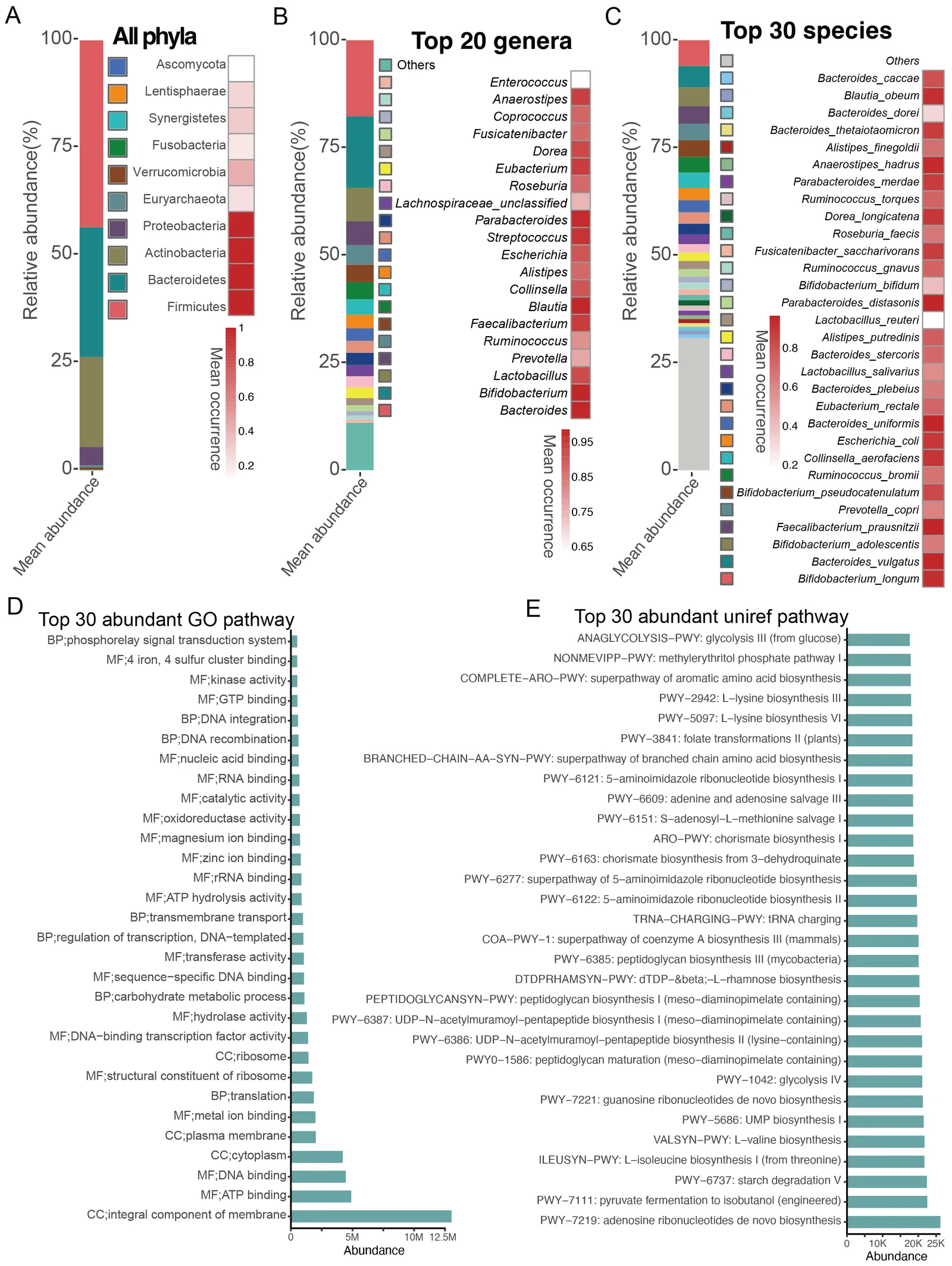
Taxonomic composition and functional profiling of fecal microbiota in gastric cancer patients. **(A)** Stacked bar chart showing the mean abundance of all detected bacterial phyla across all subjects. The right-side vertical bar indicates the mean occurrence frequency for each phylum. **(B)** Stacked bar chart illustrating the relative abundance of the top 20 dominant genera, with an auxiliary bar on the right for mean occurrence frequency. **(C)** Stacked bar plot displaying the relative abundance of the top 30 prevalent species, paired with a bar representing mean occurrence rate for each species. **(D)** Horizontal bar graph presenting the top 30 abundant Gene Ontology (GO) functional annotations, grouped into Biological Process (BP), Molecular Function (MF), and Cellular Component (CC). The horizontal axis represents pathway abundance values. **(E)** Horizontal bar chart of the top 30 enriched UniRef metabolic pathways; the horizontal axis corresponds to pathway abundance.

### 3.3 Analysis of microbial community characteristics associated with tumor staging

We further examined alterations in the composition of the gastric microbiota in GC (**Supplementary Table S9**). The flattened rarefaction curves confirmed that the sequencing depth is enough for taxonomic analysis (**Supplementary Figure 7**, **Supplementary Table S2**). Non-metric multidimensional scaling (NMDS) based on the Bray-Curtis distance matrix was performed at the genus level to evaluate differences in microbial community structure among groups II, III and IV. The NMDS ordination showed large overlaps of confidence ellipses across group II, group III and group IV. Most samples were mixed in the central area of the ordination space, while only a small number of samples from group II and group IV scattered outside the main cluster. No complete separation of clusters was observed among the three groups. Samples from different groups intersected along the MDS1 and MDS2 axes, suggesting that the overall differences in genus-level microbial community structure among group II, III and IV were not significant. During gastrectomy sampling, we collected environmental blank swabs (operating table surface, surgical glove exterior, empty sterile tube blanks) as parallel negative controls, extracted DNA following identical tissue protocols. However, all blank samples yielded total DNA <0.2 μg, failing library construction and sequencing, consistent with low-biomass gastric tissue characteristics.

β-Diversity analysis: Principal coordinate analysis (PCoA) based on genus-level Bray–Curtis distance showed that PCoA1 explained 22.1% of community variation and PCoA2 explained 16.3%, cumulatively accounting for 38.4% of microbial community differences. PERMANOVA analysis showed that tumor stage had a statistically significant yet modest effect on microbial community structure (*R*²=0.039, *P* = 0.032), explaining approximately 3.9% of total variation. While these tests indicate statistically significant differences across tumor stages (II-IV), the small effect sizes suggest that tumor stage is one of multiple co-regulating factors rather than a dominant driver of community restructuring (**Figure 2A**).

α-Diversity analysis: diversity indices at both the species level (Species_Shannon, Species_Simpson, Species_InSimpson) and the genus level (Genus_Shannon, Genus_Simpson, Genus_InSimpson) were calculated to reflect community richness and evenness. Overall, α-diversity indices did not show consistent increasing or decreasing trends across tumor stages (II, III, IV; Stage I data were not individually annotated), suggesting that microbial richness and evenness did not exhibit a uniform pattern of change during tumor progression (**Figure 2B**; **Supplementary Table S7**).

**Figure 2:**
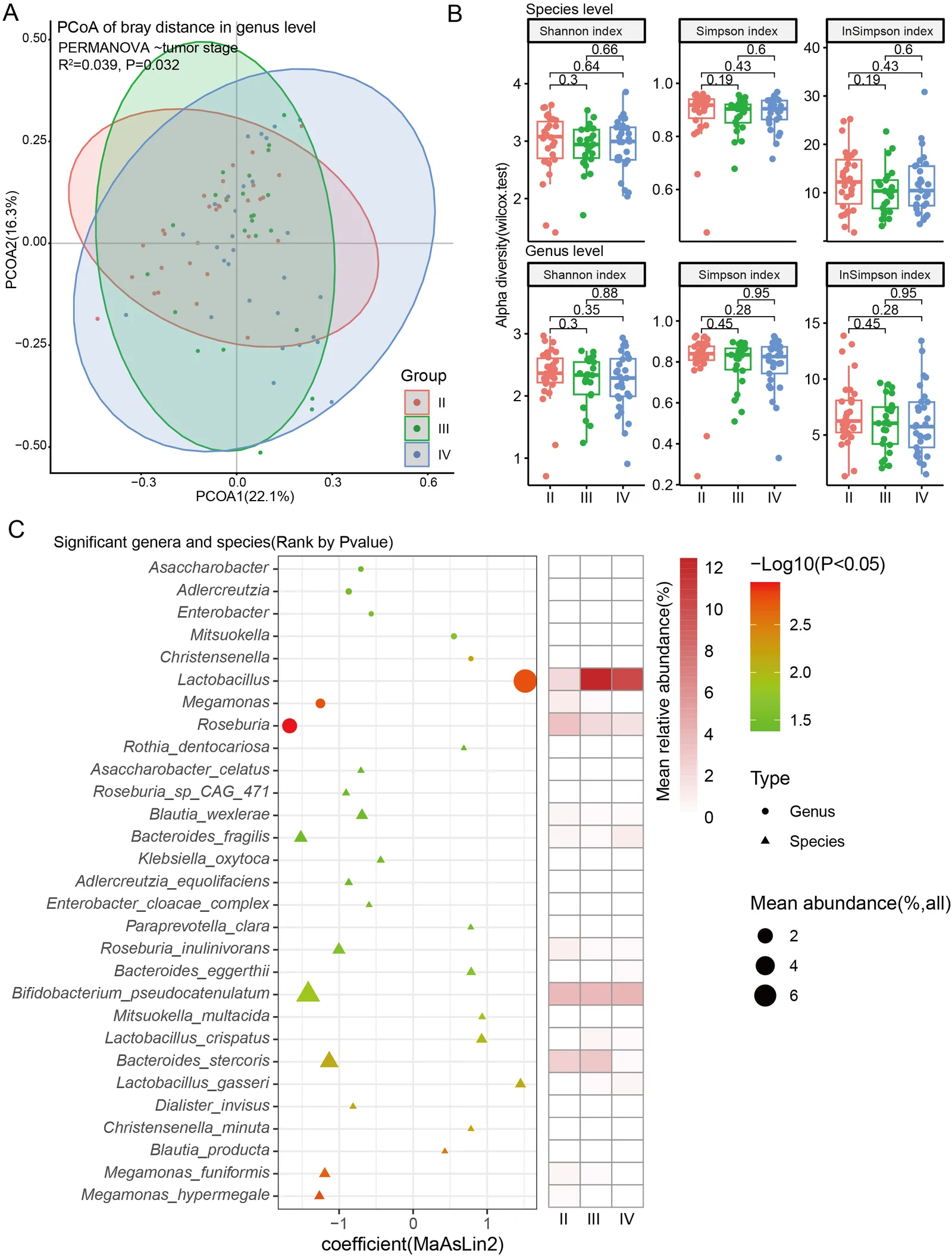
Microbial community variations stratified by AJCC pathological tumor stages. **(A)** PCoA ordination based on genus-level Bray-Curtis dissimilarity. PCoA1 explains 22.1% and PCoA2 explains 16.3% of total community variance. PERMANOVA results:*R*^2^ = 0.039, *P* = 0.032. Samples are colored by tumor stage (II-IV). **(B)** Boxplots of α-diversity indices (Species Shannon, Species Simpson, Species Inverse Simpson; Genus Shannon, Genus Simpson, Genus Inverse Simpson) across tumor stages. **(C)** Three-panel heatmap showing stage-differential genera and species derived from MaAsLin2 analysis. Left panel: -log_10_(*P* < 0.05); middle panel:mean abundance(%); right panel: mean occurrence frequency of each taxon.

We applied MaAsLin2 to identify bacterial genera and species whose relative abundances were significantly associated with tumor stage.(**Figure 2C**; **Supplementary Table S6**). Several genera exhibited strong stage-dependent abundance changes, including *Roseburia*, *Megamonas*, *Lactobacillus, Asaccharobacter, Adlercreutzia, Enterobacter, Mitsuokella*, and *Christensenella*. Among them, *Roseburia* showed a large negative regression coefficient, suggesting its abundance tended to decrease with advancing tumor stage. By contrast, *Lactobacillus* and *Enterobacter* showed positive coefficients, indicating enrichment at higher stages, while *Megamonas* presented a negative coefficient, indicating decreasing abundance at higher tumor stages.At the species level, multiple stage-associated species were detected, such as *Roseburia* sp. *CAG_471, Blautia wexlerae, Bacteroides fragilis, Adlercreutzia equolifaciens, Enterobacter cloacae-complex, Paraprevotella clara, Roseburia inulinivorans, Bacteroides eggerthii, Bifidobacterium pseudocatenulatum, Mitsuokella multacida, Lactobacillus crispatus, Bacteroides stercoris, Lactobacillus gasseri, Dialister invisus, Christensenella minuta, Blautia producta*, and *Megamonas hypermegale*. Some taxa, e.g., *Bifidobacterium pseudocatenulatum* and *Mitsuokella multacida*, displayed obvious shifts in mean relative abundance across tumor-stage subgroups according to the auxiliary heatmap. Collectively, these results demonstrated that both gut bacterial genera and species underwent stage-specific abundance alterations during gastric cancer progression.

### 3.4 Differential abundance of dominant microbial taxa in different tumor stages

#### 3.4.1 Phylum-level dominant taxa and abundance changes across stages (all phyla, P)

##### 3.4.1.1 Core dominant phyla

Absolute dominant phyla: *Firmicutes* and *Bacteroidetes* were dominant across all stages, with combined relative abundances exceeding 75%. *Firmicutes* showed the highest abundance (approximately 40%–50%), followed by *Bacteroidetes* (approximately 30%–35%), consistent with the classic “*Firmicutes–Bacteroidetes*” dominant structure of the human gut microbiota.
Major minor phyla: *Actinobacteria*, *Proteobacteria*, *Verrucomicrobia*, *Fusobacteria*, *Synergistetes*, *Euryarchaeota*, *Ascomycota*, *Lentisphaerae*, etc., with relative abundances ranging from 0% to 25%, constituted secondary community components.
Stability of dominant phyla: Relative abundances of *Firmicutes* and *Bacteroidetes* remained overall stable across Stages II–IV, suggesting no fundamental restructuring during tumor progression.
Fluctuations in minor phyla: Minor phyla such as *Actinobacteria* and *Proteobacteria* exhibited modest fluctuations without clear linear trends. (**Supplementary Figure 2A**).

#### 3.4.2 Genus-level dominant taxa and abundance changes across stages top 30 genera, P < 0.05, MaAsLin2)

Core dominant genera, including *Bacteroides, Blautia, Faecalibacterium, Ruminococcus, Bifidobacterium, Streptococcus, Escherichia, Collinsella*, and *Alistipes*, exhibited relatively high mean abundances across tumor stages. Three genera, including *Megamonas*, *Roseburia* and *Lactobacillus*, were marked with asterisks, denoting significant abundance differences across tumor stages (P < 0.05, MaAsLin2). Other key genera comprised *Parabacteroides, Dorea, Coprococcus, Fusicatenibacter, Anaerostipes*, and *Lachnospiraceae*_*unclassified*.

Core genera such as *Bacteroides, Blautia*, and *Faecalibacterium* maintained relatively stable mean abundances across stage II, III and IV. By contrast, Megamonas, *Roseburia* and *Lactobacillus* displayed prominent stage-related abundance fluctuations. Low-abundance genera (e.g., *Dorea, Anaerostipes*) showed larger variations among tumor subgroups, reflecting elevated microbial community heterogeneity in advanced tumor stages (**Supplementary Figure 2B**).

#### 3.4.3 Species-level dominant taxa and abundance changes across stages (top 30 species, P < 0.05)

Core dominant species: Bacteroides caccae, Bacteroides dorei, Bacteroides thetaiotaomicron, Bacteroides stercoris, Bacteroides uniformis, Bacteroides vulgatus, and Bacteroides plebeius.
MaAsLin2-significant species: Bacteroides stercoris and Bifidobacterium pseudocatenulatum were marked with asterisks, denoting significant abundance differences across tumor stages (P < 0.05, MaAsLin2).
Beneficial bacteria-related species: Faecalibacterium prausnitzii, Roseburia faecis, Lactobacillus reuteri, Lactobacillus salivarius, Bifidobacterium bifidum.
Other key species: Escherichia coli, Ruminococcus torques, Ruminococcus gnavus, Alistipes finegoldii.

Core species such as *Bacteroides caccae, Bacteroides dorei*, and *Faecalibacterium prausnitzii* maintained relatively stable mean abundances across stages II–IV. By contrast, the two MaAsLin2-significant species (*Bacteroides stercoris* and *Bifidobacterium pseudocatenulatum*) displayed prominent stage-related abundance fluctuations. Additionally, several probiotic species (e.g., *Lactobacillus reuteri*, *L. salivarius*) and opportunistic pathogens (e.g., E. coli) showed visible numerical changes across stages, though these did not reach statistical significance in MaAsLin2 testing. Low-abundance species (e.g., *Dorea longicatena, Anaerostipes hadrus*) showed larger variations among subgroups, reflecting elevated community heterogeneity in advanced stages (**Supplementary Figure 2C**).

### 3.5 Functional characteristics of the gastric cancer microbiome across tumor stages

To characterize the functional consequences of microbiota remodeling during gastric cancer progression, we applied MaAsLin2 to identify GO functional terms and UniRef metabolic pathways whose abundances were significantly associated with tumor stage (**Supplementary Figures 3**, **4**). All displayed features met the significance threshold of *P* < 0.05. Enrichment directions were interpreted based on the group-wise mean-abundance heatmap presented on the right side of each plot.

GO functional profiles (**Supplementary Figure 3**). A broad spectrum of GO terms exhibited significant stage-dependent abundance patterns. Features with positive MaAsLin2 coefficients (enriched in Stage II relative to advanced stages) included molecular functions related to DNA repair and maintenance, such as single-strand selective uracil DNA N-glycosylase activity, 8-oxo-7,8-dihydroguanine DNA N-glycosylase activity, purine-specific mismatch base pair DNA N-glycosylase activity, and RNA strand annealing activity. Additional Stage II-associated functions comprised aminoacyl-tRNA synthesis (asparaginyl-tRNA synthase activity), protein complex assembly (chaperone-mediated protein complex assembly), and diverse metabolic enzyme activities including hydroxymethylglutaryl-CoA synthase and mannosylglycoprotein endo-beta-mannosidase activities. Cell-envelope-associated terms, including lipoteichoic acid biosynthetic process, Gram-positive-bacterium–type cell wall, and external side of cell wall, also showed positive coefficients, as did carbohydrate utilization, nucleoside deoxyribosyltransferase activity, and several host-interaction–related terms such as modulation by symbiont of host cellular process, adhesion of symbiont to host epithelial cell, laminin binding, and P-type proton-exporting transporter activity.

Conversely, negative coefficients (indicating enrichment in Stage IV) were observed for protein modification processes such as protein deamination and protein demethylation, aspartate racemase activity, D-aspartate oxidase activity, and UDP-N-acetylglucosamine 6-dehydrogenase activity. The statistical significance of these associations, represented by −Log_10_(P), ranged from approximately 1.5 to 3.5, with mean abundances varying considerably across terms.

Metabolic pathway profiles (**Supplementary Figure 4**). At the pathway level, Stage II–enriched features (positive coefficients) were dominated by carbohydrate and energy metabolism, including lactose and galactose degradation (LACTOSECAT-PWY), glycolysis (PWY-5484, PWY-1042), anaerobic energy metabolism (PWY-7384), sucrose degradation (PWY-5384), L-glutamate and L-glutamine biosynthesis (PWY-5505), and the Calvin-Benson-Bassham cycle (CALVIN-PWY). Aromatic and polar amino acid biosynthesis pathways, such as the superpathway of aromatic amino acid biosynthesis (COMPLETE-ARO-PWY), L-tryptophan biosynthesis (TRPSYN-PWY), L-lysine biosynthesis (PWY-2941), L-ornithine biosynthesis (GLUTORN-PWY), and superpathway of heme b biosynthesis from glycine (PWY-5920), also showed positive associations. Furthermore, several nucleotide metabolism pathways, including pyrimidine deoxyribonucleotides *de novo* biosynthesis (PWY-6545, PWY-7184, PWY0-166), inosine-5-phosphate biosynthesis (PWY-7234), and queuosine biosynthesis (PWY-6700), were enriched in earlier stages, alongside the TCA cycle (REDCITCYC) and mevalonate pathway (PWY-922).

In contrast, Stage IV–enriched pathways (negative coefficients) included pyruvate fermentation to hexanol (PWY-6863),myo-/chiro-/scyllo-inositol degradation (PWY-7237), and phosphopantothenate biosynthesis (PANTO-PWY). Coenzyme and cofactor metabolism pathways, including coenzyme A biosynthesis (PANTOSYN-PWY), adenosylcobalamin salvage (COBALSYN-PWY), and thiamine diphosphate salvage (PWY-6897), displayed negative coefficient distributions, as did putrescine biosynthesis (PWY-6305), CMP-sialic acid biosynthesis (PWY-6145), and D-galacturonate degradation (GALACTUROCAT-PWY). Effect sizes ranged from approximately −1.0 to +0.5, with −Log_10_(P) values between 1.5 and 3.0. High-abundance pathways such as glycolysis, pyrimidine biosynthesis, and aromatic amino acid biosynthesis were characterized by large mean abundance circles.

### 3.6 Correlation analysis between fecal microbiota and intratumoral microbiota in gastric cancer

In this study, we constructed cross-compartment taxon abundance associations between gastric and fecal microbiota by calculating correlations using the SparCC and Spearman analysis (**Supplementary Tables S12**–**14**). **Figure 3** presents a Spearman correlation heatmap illustrating associations between fecal microbial species and cancer. The analysis focuses on correlations between high-prevalence fecal bacteria (prevalence > 0.5), cancer-associated bacteria (prevalence > 0.2), and *H. pylori*. The results reveal potential links between microbial community composition and cancer risk based on correlation coefficients and statistical significance.

**Figure 3:**
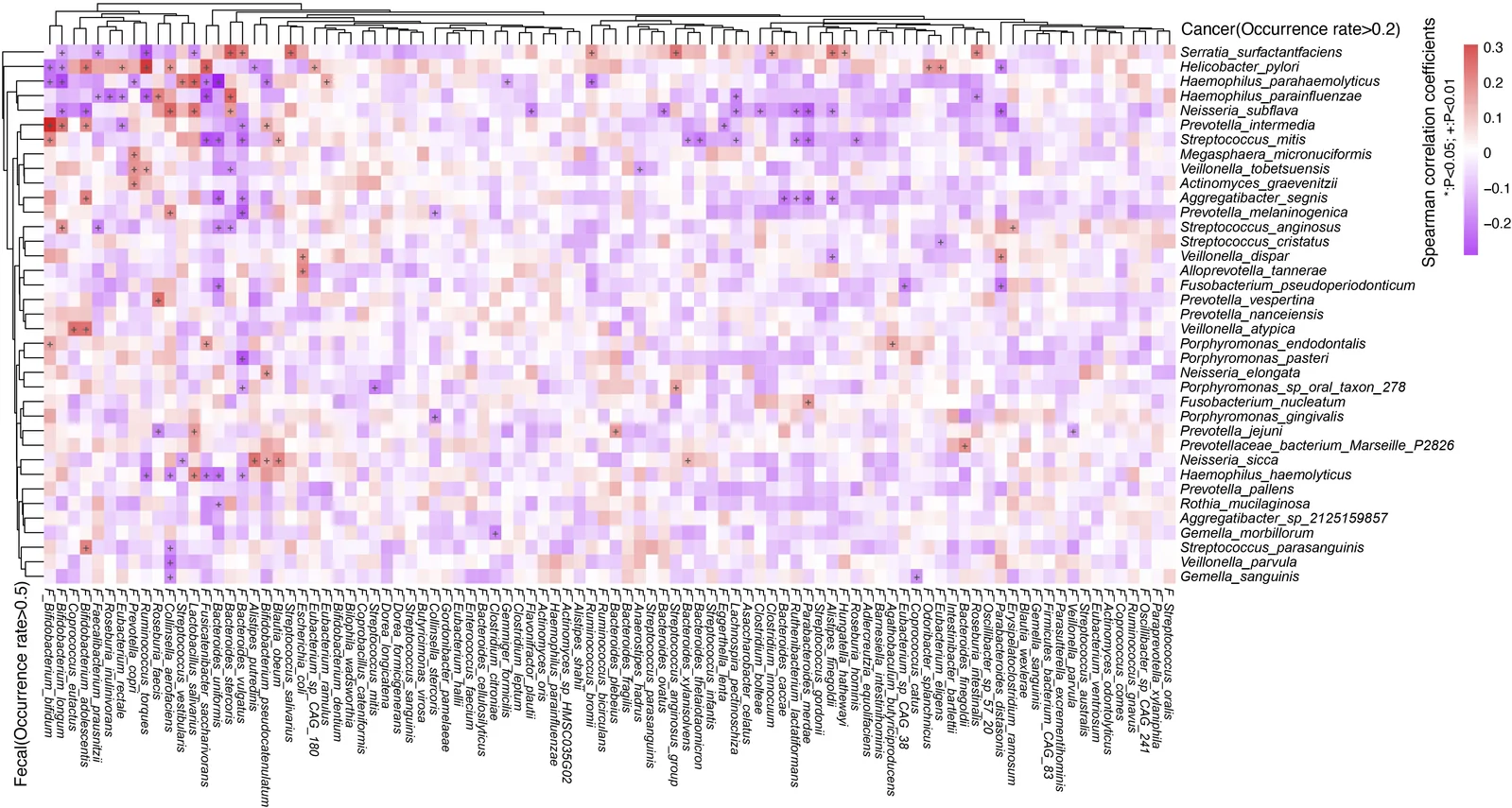
Spearman correlation heatmap between fecal and intratumoral microbial species. Heatmap visualizing pairwise Spearman correlation coefficients between high-prevalence fecal taxa (occurrence rate>0.5, vertical axis) and intratumoral microbial species with occurrence rate >0.2 (horizontal axis), including *Helicobacter pylori*. Correlation coefficient range: −0.2 ~ 0.3. Statistical markers: +*P* < 0.01.

High-Prevalence Fecal Bacteria (Fecal, Prevalence > 0.5): Over 80 gut bacterial species are included, encompassing core members of the gut microbiota such as those belonging to the phyla *Firmicutes* (e.g., *Faecalibacterium prausnitzii*, *Roseburia intestinalis*), *Bacteroidetes* (e.g., *Bacteroides thetaiotaomicron*, *Prevotella copri*), and *Actinobacteria* (e.g., *Bifidobacterium bifidum*, *Actinomyces oris*). These taxa represent dominant bacterial populations with a prevalence exceeding 50% in the study cohort.

Cancer-Associated Bacteria (Cancer, Prevalence > 0.2): This group comprises 30 bacterial species associated with cancer development, predominantly of oral origin. Representative taxa include *Fusobacterium nucleatum*, *Porphyromonas gingivalis*, *Streptococcus anginosus*, and *Helicobacter pylori*. The prevalence of these species exceeds 20% in cancer samples.

Statistical Metrics: The Spearman correlation coefficient (range: −0.2 to 0.3) was used to assess associations between bacterial taxa. An asterisk (*) indicates *P* < 0.05 (significant correlation), and a plus sign (+) indicates *P* < 0.01 (highly significant correlation).

**Figure 4** presents a Spearman correlation heatmap depicting the relationships between paracancerous tissue–associated bacteria and fecal microbiota. The analysis focuses on associations between “High-Prevalence Fecal Bacteria (Prevalence > 0.5)” and “Paracancerous Tissue–Associated Bacteria (Prevalence > 0.2),” with particular emphasis on *Helicobacter pylori*. Correlation coefficients (−0.3 to 0.3) and statistical significance markers (*P* < 0.05, +*P* < 0.01) are used to characterize cross-compartment taxon abundance association matrix within the paracancerous microenvironment.

**Figure 4:**
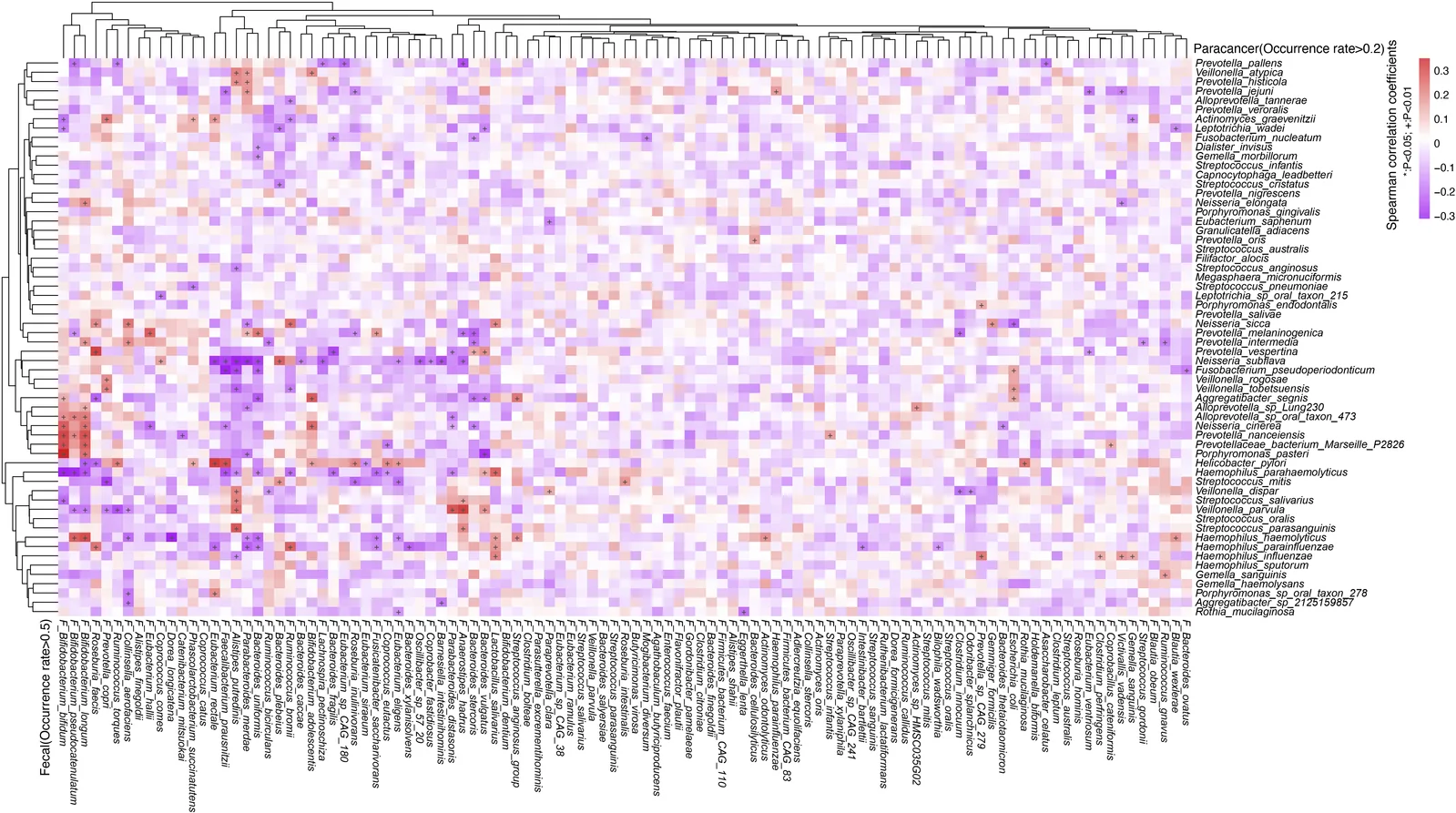
Spearman correlation heatmap between fecal and paratumor mucosal microbial species. Heatmap of Spearman correlation values for cross-compartment associations: vertical axis = high-prevalence fecal taxa (occurrence rate >0.5); horizontal axis = microbial species detected in paratumor mucosal tissues (occurrence rate >0.2). Correlation range: −0.3 ~ 0.3. Significance symbols: +*P* < 0.01.

I. Explanation of the Figure’s Core Elements 1. Microbial Grouping and Screening Criteria

High-Prevalence Fecal Bacteria (Fecal, Prevalence > 0.5): This group includes over 80 dominant gut bacterial species representing major phyla such as *Firmicutes*, *Bacteroidetes*, and *Actinobacteria*. These taxa represent stably colonizing microorganisms with prevalence exceeding 50% in the study population. Representative taxa include beneficial bacteria (*Faecalibacterium prausnitzii*, *Roseburia intestinalis*, *Bifidobacterium* spp.), conditional pathogens (*Escherichia coli*, *Clostridium perfringens*, *Bacteroides fragilis*), and commensal species (*Bacteroides vulgatus*, *Bacteroides ovatus*).

Paracancerous Tissue–Associated Bacteria (Paracancer, Prevalence > 0.2): This group comprises 30 bacterial species enriched in paracancerous tissues, predominantly of oral origin. Core taxa include known cancer-associated or procarcinogenic species such as *Helicobacter pylori*, *Fusobacterium nucleatum*, *Porphyromonas gingivalis*, and *Streptococcus anginosus*, as well as oral commensals including *Haemophilus parainfluenzae*, *Veillonella* spp. (e.g., *V. parvula*, *V. atypica*), and *Neisseria* spp. (e.g., *N. sicca*, *N. elongata*).

Statistical Metrics: The Spearman correlation coefficient (r) reflects the strength of association (range: −0.3 to 0.3). An asterisk (*) denotes *P* < 0.05, and a plus sign (+) denotes *P* < 0.01.

**Supplementary Figure 5** presents a phylum-level Spearman correlation heatmap comprising “Module A (Cancer-Associated Bacteria vs. Fecal Microbiota)” and “Module B (Paracancerous Tissue–Associated Bacteria vs. Fecal Microbiota).” The analysis focuses on phylum-level associations between “Cancer/Paracancerous Tissue–Associated Bacteria (Prevalence > 0.1)” and “Fecal Microbiota (Prevalence Rate > 0.1)”. Correlation coefficients and statistical significance tests were utilized to elucidate the characteristics of interactions between the phylum-level structure of microbial communities and the tumor microenvironment.

I. Explanation of the Figure’s Core Elements 1. Phylum-Level Microbial Grouping and Screening Criteria

Cancer-Associated Bacteria (Cancer, Prevalence > 0.1): This group includes six core bacterial phyla with prevalence exceeding 10% in cancer tissues, including *Proteobacteria, Bacteroidetes, Fusobacteria, Candidatus_Saccharibacteria, Firmicutes*, and *Actinobacteria*.

Paracancerous Tissue–Associated Bacteria (Paracancer, Prevalence > 0.1): This group comprises six bacterial phyla, largely overlapping with cancer-associated phyla. Core phyla such as *Firmicutes, Bacteroidetes, Candidatus_Saccharibacteria, Proteobacteria, Fusobacteria*, and *Actinobacteria* remain prominent.

Fecal Microbiota (Fecal, Prevalence > 0.1): This group encompasses nine dominant bacterial phyla, including *Firmicutes*, *Bacteroidetes*, *Actinobacteria*, and *Proteobacteria*, which collectively represent the major constituents of the gut microbiome.

Statistical Metrics: Correlation coefficients range from −0.1 to 0.2 for Module A and from −0.3 to 0.2 for Module B. Larger absolute values indicate stronger correlations. Statistical significance is indicated by * (P < 0.05) and + (P < 0.01).

**Supplementary Figure 6** presents a dual-module Spearman correlation heatmap including “Module A (Cancer-Associated Bacteria vs. Fecal Microbiota)” and “Module B (Paracancerous Tissue–Associated Bacteria vs. Fecal Microbiota).” The analysis focuses on genus-level associations between cancer/paracancerous tissue–associated bacteria (occurrence rate > 0.1) and fecal microbiota (occurrence rate > 0.1), with particular emphasis on *H. pylori*. Correlation coefficients (−0.3 to 0.4) and statistical significance indicators (*P* < 0.05, +*P* < 0.01) are used to characterize interaction patterns.

I. Analysis of Core Elements Microbial Grouping and Screening Criteria

Cancer-Associated Bacteria (Cancer, Occurrence Rate > 0.1): This group includes 24 bacterial genera, primarily composed of oral-derived procarcinogenic taxa. Representative genera include: Class I carcinogens/procarcinogens such as *Helicobacter, Fusobacterium, Porphyromonas*, and *Prevotella*; Oral commensals/opportunistic pathogens, such as S*treptococcus, Haemophilus, Veillonella*, and *Actinomyces;* and other relevant bacteria, such as *Pseudomonas, Serratia, and Campylobacter*. Paracancerous tissue-associated bacteria (Paracancer, Occurrence Rate > 0.1): This group includes 22 bacterial genera and largely overlaps with cancer-associated taxa, while also incorporating additional genera such as *Filifactor*. Core procarcinogenic genera, including *Helicobacter*, *Fusobacterium*, and *Porphyromonas*, remain dominant.

Fecal Microbiota (Fecal, Occurrence Rate > 0.1): This group comprises more than 50 bacterial genera representing major gut phyla, including *Firmicutes*, *Bacteroidetes*, *Actinobacteria*, and *Proteobacteria*. Representative genera include beneficial taxa (*Bifidobacterium*, *Faecalibacterium*, *Roseburia*, *Ruminococcus*), opportunistic pathogens (*Escherichia*, *Klebsiella*, *Clostridium*, *Desulfovibrio*), and commensal genera (*Bacteroides*, *Prevotella*, *Veillonella*).

Statistical Indicators Correlation coefficient range for Module A: -0.3~0.4 (higher values indicate stronger positive correlations, lower values indicate stronger negative correlations); Correlation coefficient range for Module B: -0.3~0.4; Significance markers: * (P<0.05, significant correlation), + (P<0.01, highly significant correlation).

## 4 Discussion

### 4.1 Stability and specific changes in the composition of microbial communities

This study systematically analyzed the compositional characteristics of fecal microbiota at the phylum, genus, and species levels in cancer and paracancerous states, demonstrating that the community exhibits a core structural pattern characterized by “dominance at the phylum level and diversity at the genus/species level.” Firmicutes and Bacteroidetes, as the absolute dominant phyla (collectively accounting for over 75%), maintained structural stability across tumor stages I–IV and in paracancerous tissues. This observation is consistent with the classical compositional features of the human gut microbiota (Qin et al., 2010; Bäckhed et al., 2005) and supports the concept of “core microbiota resilience” proposed by Feng et al (Feng et al., 2022), indicating that core microbial phyla preserve the fundamental ecological framework of the gut despite tumor-associated perturbations, thereby sustaining essential host metabolic functions.

The distribution patterns of dominant genera (e.g., *Bacteroides*, *Bifidobacterium*, *Lactobacillus*) and species (e.g., *Faecalibacterium prausnitzii*, *Fusobacterium nucleatum*) further support the principle of “conservation of core microbiota” (Faith et al., 2013; Zhang et al., 2024), suggesting that these microbes contribute to host physiological stability by maintaining intestinal microenvironmental homeostasis. Tumor stage exhibited marginally significant associations with overall microbial community structure (PERMANOVA, *R*² = 0.039, *P* = 0.032) but only accounts for ~3.9% of total microbial compositional variation, confirmed by PERMDISP test ruling out uneven sample dispersion as a confounding factor. Tumor progression represents only one of multiple co-regulating clinical variables including H. pylori status and chemotherapy history.

However, clear stage-specific clustering was not observed (NMDS stress = 0.09), indicating that tumor progression influences microbial composition in a graded rather than discrete manner. This finding is consistent with the results of Zhang et al (Zhang et al., 2018), who reported that although tumor stage significantly correlates with microbial β-diversity, community differentiation is also shaped by host immune status and therapeutic interventions. Together, these findings support the concept of “multifactorial co-regulation of the tumor microbiome” proposed by Galeano Niño et al (Galeano Niño et al., 2022).

At finer taxonomic resolution, differential taxa across stages—including the phylum *Euryarchaeota* and genera such as *Roseburia* and *Lactobacillus*—exhibited non-linear abundance patterns, further supporting the above conclusions. Moreover, the number of associated species in the paracancerous state (45 species) increased by 61% compared with the cancer group (28 species), with the occurrence threshold set at 0.1, reflecting greater microbial heterogeneity in paratumor mucosal tissue. The dispersed distribution of late-stage (Stage IV) samples further indicated increased inter-individual variability in microbiota composition, consistent with observations by Kartal et al (Kartal et al., 2022) in pancreatic cancer. These findings suggest that interindividual heterogeneity should be prioritized when considering microbial biomarkers in advanced cancer.

### 4.2 Functional remodeling: the central role of metabolic pathways in tumor progression

Functional pathway analysis revealed that the core microbial functions in cancer and paracancerous states are concentrated in three major modules: carbohydrate metabolism, amino acid biosynthesis, and nucleotide metabolism. Enrichment of molecular functions related to catalytic activity and transferase activity, together with significant differences in UniRef pathways such as glycolysis and aromatic amino acid biosynthesis (*P* < 0.01), indicates that microbial communities participate in tumor progression through metabolic reprogramming. These findings are consistent with the “microbiome–cancer” framework proposed by Sepich-Poore et al (Sepich-Poore et al., 2021), which emphasizes metabolites as key mediators of microbe–host interactions.

Stage III patients harbored the largest number of differentially remodeled microbial metabolic pathways. However, cross-sectional observational data cannot establish causal temporal metabolic shifts during sequential GC progression. Full statistical metrics including log2 fold change, effect size and FDR-adjusted Q-values are provided in **Supplementary Tables S10** and **S11**, with bubble plots annotated with significance thresholds in supplementary figures. This observation aligns with findings by Wang et al (Wang et al., 2022), who reported pronounced metabolic shifts during mid-stage colorectal cancer progression. At this stage, microbial communities have been hypothesized to support tumor growth by enhancing energy supply through upregulation of glycolytic pathways and amino acid biosynthesis, including branched-chain amino acid metabolism. Cross-stage comparisons further revealed that the most significant functional divergence occurred among Stages II, III and IV, involving eight highly significant UniRef pathways related primarily to carbohydrate and aromatic amino acid metabolism. This pattern is consistent with the concept of progressively escalating metabolic demand during tumor development (Mager et al., 2020). The pattern of functional specialization and stabilization observed in advanced stages (III/IV) may reflect the microenvironmental adaptation process as the tumor transitions from a local lesion to a systemic influence.

Notably, the coexistence of extensive remodeling in high-abundance core metabolic pathways and selective alterations in low-abundance pathways highlights a dual regulatory pattern of microbial function. This observation supports the hypothesis proposed by Torres-Maravilla et al (Torres-Maravilla et al., 2021) that microbial functional redundancy and specificity coexist during carcinogenesis. In the paracancerous state, a broader spectrum of functionally associated pathways was observed, accompanied by a reduction in non-significant associations. In particular, negative correlations involving probiotic-associated metabolic pathways, such as short-chain fatty acid biosynthesis, were more pronounced, suggesting that functional dysregulation of the microbiota begins prior to malignant transformation and may represent an early target for preventive intervention.

### 4.3 Microbial correlation patterns in tumor and paratumor tissues

This study constructed a cross-hierarchical (phylum–genus–species) microbe–cancer association profile, clearly distinguishing the microbiota characteristics of cancerous and paracancerous (precancerous) states and revealing a core pattern of “pathogen enrichment and beneficial bacteria depletion.”

#### 4.3.1 Risk-associated microbes

*F. nucleatum* exhibited the strongest association in the paracancerous group (r ≈ 0.52), followed by the cancer group (r ≈ 0.75). As a well-established tumor-promoting bacterium, *F. nucleatum* promotes tumorigenesis through activation of the NF-κB inflammatory pathway and enhancement of tumor cell invasion and metastasis (Bullman et al., 2017; Wu et al., 2025). Its persistent enrichment in colorectal tumors has been consistently reported, reinforcing its role in cancer progression (Bullman et al., 2017) and its correlation with tumor progression.

A strong association of *H*. *pylori* with gastric and colorectal cancers was observed (r≈0.68), further supporting its classification as a Group I carcinogen by the World Health Organization (WHO, 2004). Its carcinogenic mechanisms, including induction of DNA damage and chronic inflammation, have been extensively documented (WHO, 2004; Polk et al., 2010).

Notably, this study identified a significant positive correlation for the genus *Tannerella* in the paracancerous group (r ≈ 0.48). Combined with previous findings by Flemer et al. (2018) demonstrating associations between oral microbiota and colorectal cancer, these results suggest that oral-derived bacteria may translocate through the oral–gut axis and contribute to precancerous lesion development. This observation warrants further validation in larger cohorts.

#### 4.3.2 Microbes associated with protective effects

Short-chain fatty acid–producing bacteria, including *Roseburia* and *Faecalibacterium*, exhibited significantly stronger negative correlations in the paracancerous group (r≈-0.55, -0.52, respectively) than in the cancer group. The butyrate produced by these taxa is known to inhibit tumor cell proliferation through histone deacetylase (HDAC) inhibition (Pryde et al., 2012). The anti-inflammatory properties of *Faecalibacterium prausnitzii* have also been well documented (Sokol et al., 2008).

Consistent with these observations, the negative association of *Bifidobacterium* supports its protective role in maintaining intestinal barrier integrity and regulating immune homeostasis (O'Callaghan and van Sinderen, 2016). The pronounced depletion of beneficial bacteria in the paracancerous state aligns with the dysbiosis theory of “loss of protectors preceding invader colonization” proposed by Wirbel et al (Wirbel et al., 2019) in their meta-analysis examining colorectal cancer. The depletion of beneficial bacteria, such as *Roseburia* and *Faecalibacterium* leads to weakened intestinal barrier function and reduced anti-inflammatory capacity, leading to favorable conditions for the colonization of tumor-promoting bacteria and carcinogenesis (Eysha et al., 2026). Therefore, this ecological shift may represent a candidate indicator for future validation in controlled cohorts, but cannot serve as an early warning biomarker in the absence of non-cancer controls.

Phylum-level analysis further demonstrated that the combined enrichment of *Fusobacteria*, *Proteobacteria*, and *Bacteroidetes*, together with the depletion of *Firmicutes* and *Actinobacteria*, was significantly strengthened in the paracancerous group.At the genus level, *Fusobacterium* (r ≈ 0.75) and *Helicobacter* (r ≈ 0.68) were most strongly associated with cancer, whereas *Roseburia* (r ≈ −0.55) and *Tannerella* (r ≈ 0.48) showed greater relevance in the paracancerous state. Together, these findings support a hierarchical “phylum–genus–species” biomarker framework, consistent with the microbiome hierarchical biomarker model” proposed by de Vos and de Vos (de Vos and de Vos, 2012), enhancing the precision and flexibility of clinical application.

### 4.4 Clinical significance and future directions

The proposed candidate microbial taxon signatures associated with GC tumor progression provides an integrated framework for early detection, risk stratification, and prognostic evaluation of tumor development. Similarly, marked enrichment of *H. pylori* and *F. nucleatum* in cancerous tissues may indicate active tumor–microbe interactions with diagnostic and therapeutic relevance (Zeller et al., 2014; Bautista et al., 2025). The observed enrichment of oral-origin taxa such as *Tannerella* and *Porphyromonas* further supports the involvement of the oral–gut axis in tumorigenesis and highlights potential targets for microbiome-based intervention.

Future research should prioritize three directions: (1) validation of these microbial biomarkers in larger, independent cohorts to assess predictive accuracy and robustness; (2) mechanistic studies using germ-free or gnotobiotic models to elucidate how key taxa and their metabolites (e.g., amino acid–derived metabolites) influence carcinogenic processes; and (3) development of personalized microbiome-based preventive or therapeutic strategies based on microbial biomarkers, such as modulation of the gut microbiota including high-risk populations through prebiotics, probiotics, or precise antibiotic therapy to achieve cancer prevention or adjuvant treatment.

### 4.5 Study limitations

1. Single-center cross-sectional observational design without healthy volunteers, atrophic gastritis or precancerous lesion control groups; all observed microbial shifts only reflect intra-cohort variation and cannot be confirmed as GC-specific signatures.
2. No independent external cohort, machine learning or ROC analysis to verify predictive performance of candidate microbial taxon signatures; these taxa cannot be applied for clinical diagnosis or early warning currently.
3. As noted by Fierer et al. (2025), negative controls should be considered in metagenomic sequencing. Regrettably, we did not include such controls in our library construction and sequencing due to the very low total DNA content. Although we could not include true negative controls due to external sequencing logistics, the application of statistical decontamination tools-Decontam-strengthens the validity of the observed biological signals. In our upcoming multi-center sampling study, we plan to include negative controls and further optimize the procedures for micro-library construction and sequencing”.

## 5 Conclusions

In summary, gut, tumor and paratumor mucosal microbial communities of GC patients maintain stable core phylum composition, but present stage-specific species-level remodeling. This study systematically describes stable core phylum-level microbial composition alongside stage-specific species abundance remodeling and metabolic functional variation across fecal, intratumoral and paratumor mucosal compartments among 85 gastric cancer patients from Northwest China. Cross-compartment taxon abundance associations within this single GC cohort were characterized, and multi-layer intra-cohort taxonomic correlation profiles were constructed. All observed microbial shifts are only candidate correlative signatures linked to GC progression within this patient group; without non-cancer control cohorts, these taxa cannot serve as gastric cancer diagnostic or precancer early-warning indicators. Further multi-center case-control cohort research is required to validate the clinical relevance of these microbial taxonomic features.

## Data Availability

The datasets presented in this study can be found in online repositories. The names of the repository/repositories and accession number(s) can be found in the article/**Supplementary Material**.

## Supplementary material

Supplementary Figure 1 NMDS ordination of genus-level microbiota profiles. Non-metric multidimensional scaling (NMDS) plot generated with Bray-Curtis distance. Each dot represents one sample; ellipses and colors distinguish three tumor stages (II-IV). X-axis = MDS1, Y-axis = MDS2.

Supplementary Figure 2 Stage-stratified taxonomic relative abundance profiles. **(A)** Stacked bar chart of mean phylum-level relative abundance across tumor stages II-IV; phyla marked with * show inter-stage differences (*P* < 0.05). **(B)** Stacked bar plot of the top 30 genera stratified by tumor stage; genera with significant stage variation labeled *. **(C)** Stacked bar graph of the top 30 species across three tumor stages; species with (*P* < 0.05) ifferences are annotated with *. Vertical axis = relative abundance (%).

Supplementary Figure 3 Bubble plot of differentially abundant GO functional terms across tumor stages. Bubble plot visualizing GO terms with stage-associated differences identified by MaAsLin2. Vertical axis =-log_10_(*P* < 0.05), horizontal axis: MaAsLin2 regression coefficient. Bubble size indicates mean pathway abundance across all samples. Sign markers: **P* < 0.05; +*P* < 0.01. Bubble plot visualizing GO terms with stage-associated differences determined by MaAsLin2 analysis. The horizontal axis shows the MaAsLin2 regression coefficient, and the vertical axis lists individual GO term descriptions grouped into Biological Process (BP), Molecular Function (MF), and Cellular Component (CC). Bubble color encodes -log10(P<0.05), while bubble size represents the mean GO-term abundance across all samples. The right-hand heatmap panel displays mean GO-term abundance for tumor-stage comparisons (II-IV).

Supplementary Figure 4 Bubble plot of differentially enriched UniRef metabolic pathways across tumor stages. Bubble plot illustrating UniRef metabolic pathways showing stage-associated differences identified by MaAsLin2 analysis. The horizontal axis represents MaAsLin2 regression coefficient; the vertical axis lists individual annotated UniRef pathway IDs and full-length pathway descriptions. Bubble color denotes -log10(P<0.05) and bubble size corresponds to mean pathway abundance across all samples. The right-side heatmap panel shows mean pathway abundance for tumor-stage comparisons (II-IV).

Supplementary Figure 5 Phylum-level cross-compartment Spearman correlation heatmaps. **(A)** Correlation matrix: intratumor-associated phyla (occurrence rate > 0.1) versus fecal phyla (occurrence rate > 0.1). Correlation range: −0.1 ~ 0.2. **(B)** Correlation matrix: paratumor mucosa-associated phyla (occurrence rate > 0.1) versus fecal phyla (occurrence rate > 0.1). Correlation range: −0.3 ~ 0.2. Color gradient represents Spearman correlation coefficients; significance markers: **P* < 0.05; +*P* < 0.01.

Supplementary Figure 6 Genus-level cross-compartment Spearman correlation heatmaps. **(A)** Spearman correlation heatmap between tumor-associated genera (occurrence rate > 0.1, vertical) and fecal genera (occurrence rate > 0.1, horizontal). Coefficient range: −0.2 ~ 0.3. **(B)** Spearman correlation heatmap between paratumor mucosa-associated genera (occurrence rate > 0.1, vertical) and fecal genera (occurrence rate > 0.1, horizontal). Coefficient range: −0.4 ~ 0.3. Color scale indicates correlation strength; significance: **P* < 0.05; +*P* < 0.01.

Supplementary Figure 7 Rarefaction curves evaluating sequencing-depth sufficiency. Rarefaction curves were generated from six randomly selected samples. The x-axis represents the number of sampled reads, and the y-axis represents observed species-level Shannon index. The curves gradually plateau, demonstrating that the sequencing depth was adequate to capture most microbial species in the present dataset. Corresponding quantitative metrics are summarized in **Supplementary Table S2**.

Supplementary Figure 8 Venn diagram illustrating sample overlap across three sample compartments. Venn diagram showing the overlap of samples among three specimen groups: fecal (Cancer_fecal), intratumoral cancer tissue (Cancer), and paratumor mucosal tissue (Paracancer). Numbers inside each region represent sample counts in corresponding subsets.
